# On the Normed Space of Equivalence Classes of Fuzzy Numbers

**DOI:** 10.1155/2013/792484

**Published:** 2013-08-29

**Authors:** Dong Qiu, Chongxia Lu, Wei Zhang

**Affiliations:** College of Mathematics and Physics, Chongqing University of Posts and Telecommunications, Nanan, Chongqing 400065, China

## Abstract

We study the norm induced by the supremum metric on the space of fuzzy numbers. And then we propose a method for constructing a norm on the quotient space of fuzzy numbers. This norm is very natural and works well with the induced metric on the quotient space.

## 1. Introduction

Since the concept of fuzzy numbers was firstly introduced in the 1970s, it has been studied extensively from many different aspects of the theory and applications such as fuzzy topology, fuzzy analysis, fuzzy logic, and fuzzy decision making (see, e.g., [1–6]). The operations in the set of fuzzy numbers are usually obtained by the Zadeh extension principle [7–9]. In the study of algebraic structures and topological structures for fuzzy numbers, many results have been obtained (see, e.g., [10–19]).

In the classical mathematics, if *X* is a normed space with norm ||·||, it is readily checked that the formula *d*(*x*, *y*) = ||*x* − *y*||, for *x*, *y* ∈ *X*, defines a metric *d* on *X*. Thus a normed space is naturally a metric space and all metric space concepts are meaningful. However, we will show that such proposition does not hold true for the well known supremum metric on the space of fuzzy numbers. To overcome this weakness, we will consider the quotient space of fuzzy numbers up to an equivalence relation which is introduced by Mareš [20, 21] and is studied extensively by many researchers [4, 12, 22–24]. We will propose a method for constructing a norm on the quotient space of fuzzy numbers. This norm is very natural and works well with the induced metric on the quotient space.

## 2. Preliminaries

A fuzzy set of ℝ is a function *μ* : ℝ → [0,1]. For each such fuzzy set *μ*, we denote by [*μ*]^*a*^ = {*x* ∈ ℝ : *μ*(*x*) ≥ *a*} for any *a* ∈ (0,1] its *a*-level set. We define the set [*μ*]^0^ by ${\left[\mu \right]}^{0}=\bar{\left\{x\in \mathbb{R}:\mu (x)>0\right\}}$, where $\bar{A}$ denotes the closure of a set *A*. A fuzzy number *μ* is a fuzzy set with nonempty bounded closed level sets [*μ*]^*a*^ = [*μ*_*L*_(*a*), *μ*_*R*_(*a*)] for all *a* ∈ [0,1]. We denote the class of fuzzy numbers by *ℱ*. Notice that the real numbers ℝ can be imbedded in *ℱ* by defining a fuzzy number as follows

(1) $$\begin{array}{c} a\left(x\right)=\{\begin{array}{cc} 1, & \text{if}\;x=a,\\ 0, & \text{otherwise}, \end{array} \end{array}$$

for each *a* ∈ ℝ.

For any *μ*, *ν* ∈ *ℱ* and *a* ∈ ℝ, owing to Zadeh's extension principle [7–9], scalar multiplication and addition are defined for any *x* ∈ ℝ by

(2) $$\begin{array}{c} \left(a\times \mu \right)\left(x\right)=\left(a\mu \right)\left(x\right)=\{\begin{array}{cc} \mu \left(\frac{x}{a}\right), & \text{if}\;a\neq 0,\\ 0, & \text{if}\;a=0, \end{array}\\ \left(\mu +\nu \right)\left(x\right)=\underset{x_{1},x_{2}:x_{1}+x_{2}=x}{\sup}\min\left\{\mu \left(x_{1}\right),\nu \left(x_{2}\right)\right\}. \end{array}$$

For any *μ* ∈ *ℱ*, we define the fuzzy number −*μ* ∈ *ℱ* by −*μ* = (−1) × *μ*, that is, −*μ*(*x*) = *μ*(−*x*), for all *x* ∈ ℝ. Since the addition does not possess an inverse subtraction, *ℱ* is not a real vector space. We say that a fuzzy number *s* ∈ *ℱ* is symmetric [20], if

(3) $$\begin{array}{c} s\left(x\right)=s\left(-x\right), \end{array}$$

for all *x* ∈ ℝ, that is, *s* = −*s*. The set of all symmetric fuzzy numbers will be denoted by *𝒮*.

**Definition 1 (see [22])**

Let *μ*, *ν* ∈ *ℱ*. We say that *μ* is equivalent to *ν* and write *μ* ~ *ν* if and only if there exist symmetric fuzzy numbers *s*_1_, *s*_2_ ∈ *𝒮* such that

(4) $$\begin{array}{c} \mu +s_{1}=\nu +s_{2}. \end{array}$$

The equivalence relation defined above is reflexive, symmetric, and transitive [20]. Let 〈*μ*〉 denote the equivalence class containing the element *μ* and denote the set of equivalence classes by *ℱ*/*𝒮*. By the level set representations for fuzzy numbers, one can easily prove the following lemmas.

**Lemma 2 (see [25])**

For any *μ* ∈ *ℱ*, *μ* − *μ* ∈ *𝒮*.

**Lemma 3 (see [25])**

For any *μ*, *ν* ∈ *ℱ*, *μ* ~ *ν* if and only if *μ* − *ν* ∈ *𝒮*.

**Definition 4 (see [26])**

For a fuzzy number *μ*, we define a function *μ*_*M*_ : [0,1] → ℝ by assigning the midpoint of each *a*-level set to *μ*_*M*_(*a*) for all *a* ∈ [0,1], that is,

(5) $$\begin{array}{c} \mu_{M}\left(a\right)=\frac{\mu_{L}\left(a\right)+\mu_{R}\left(a\right)}{2}. \end{array}$$

**Lemma 5 (see [25])**

For any *μ*, *ν* ∈ *ℱ*, *μ* ~ *ν* if and only if *μ*_*M*_ = *ν*_*M*_.

**Lemma 6 (see [26])**

(*ℱ*/*𝒮*, +, ×) is a real vector space.

## 3. Main Results

In this section, we will give a norm structure on *ℱ*/*𝒮* which is compatible with a metric. For the family of fuzzy numbers *ℱ*, the *d*_*∞*_-metric is induced by the Hausdorff metric as [17]

(6) d∞(μ,ν)=sup⁡0≤a≤1H([μ]a,[ν]a)=sup⁡0≤a≤1max⁡{|μL(a)−νL(a)|,|μR(a)−νR(a)|},

for all *μ*, *ν* ∈ *ℱ*.

Define a function ||·|| : *ℱ* → ℝ as

(7) $$\begin{array}{c} ||\mu ||=d\left(\mu ,0\right)=\underset{0\leq a\leq 1}{\sup}\max\left\{\left|\mu_{L}\left(a\right)\right|,\left|\mu_{R}\left(a\right)\right|\right\}. \end{array}$$

**Theorem 7**

The function ||·|| in (7) is a norm on *ℱ*.

**Proof**

(i)  It is obvious that ||*μ*|| ≥ 0 for all *μ* ∈ *ℱ* and ||*μ*|| = 0 if *μ* = 0. Conversely, if *μ* ≠ 0, then we have that there exists *a*_0_ ∈ [0,1] such that [*μ*_*L*_(*a*_0_), *μ*_*R*_(*a*_0_)]≠{0}. Thus we have ||*μ*|| > 0.

(ii)  For all *μ* ∈ *ℱ* and *b* ∈ ℝ, we have

(8) ||bμ||=sup⁡0≤a≤1max⁡{|b||μL(a)|,|b||μR(a)|}=|b|||μ||.

(iii)  For all *μ*, *ν* ∈ *ℱ*, we have that

(9) ||μ+ν||=sup⁡0≤a≤1max⁡{|μL(a)+νL(a)|,|μR(a)+νR(a)|}≤sup⁡0≤a≤1max⁡{|μL(a)|+|νL(a)|,|μR(a)|+|νR(a)|}≤sup⁡0≤a≤1max⁡{|μL(a)|,|μR(a)|} +sup⁡0≤a≤1max⁡{|νL(a)|,|νR(a)|}=||μ||+||ν||.

We conclude that ||·|| is a norm on *ℱ*.

Although ||·|| is a norm on *ℱ*, the function *d* : *ℱ* × *ℱ* → ℝ induced by ||·|| as *d*(*μ*, *ν*) = ||*μ* − *ν*|| is not a metric on *ℱ*. Consequently, we get that *d*_*∞*_(*μ*, *ν*) ≠ ||*μ* − *ν*||.

**Theorem 8**

The function *d* has the following properties:

- *d*(*μ*, *ν*) ≥ 0, for any *μ*, *ν* ∈ *ℱ*;
- *d*(*μ*, *ν*) = *d*(*ν*, *μ*), for any *μ*, *ν* ∈ *ℱ*;
- *d*(*μ*, *ν*) ≤ *d*(*μ*, *ω*) + *d*(*ω*, *ν*), for any *μ*, *ν*, *ω* ∈ *ℱ*;
- *d*(*μ*, *μ*) ≠ 0, for any fuzzy number *μ* ∉ ℝ.

**Proof**

(i)  By the definition of *d*, it is obvious that *d*(*μ*, *ν*) ≥ 0, for any *μ*, *ν* ∈ *ℱ*.

(ii)  For all *μ*, *ν* ∈ *ℱ*, we have that

(10) d(μ,ν)=||μ−ν||=sup⁡0≤a≤1max⁡{|μL(a)−νR(a)|,|μR(a)−νL(a)|}=sup⁡0≤a≤1max⁡{|νL(a)−μR(a)|,|νR(a)−μL(a)|}=||ν−μ||=d(ν,μ).

(iii)  In order to prove the triangle inequality, for any fixed *a* ∈ [0,1], and for any *μ*, *ν*, *ω* ∈ *ℱ*, we only proof the following six cases. Similarly, the others can be proved.

*Case  1 ( μ*_*L*_(*a*) ≤ *ν*_*L*_(*a*) ≤ *μ*_*R*_(*a*) ≤ *ν*_*R*_(*a*) and *ω*_*L*_(*a*) ≤ *μ*_*L*_(*a*)). In this case we have that

(11) max⁡{|μL(a)−νR(a)|,|μR(a)−νL(a)|}   =|μL(a)−νR(a)| ≤|ωL(a)−νR(a)| ≤max⁡{|μL(a)−ωR(a)|,|μR(a)−ωL(a)|}    +max⁡{|ωL(a)−νR(a)|,|ωR(a)−νL(a)|}.

*Case  2 ( μ*_*L*_(*a*) ≤ *ν*_*L*_(*a*) ≤ *μ*_*R*_(*a*) ≤ *ν*_*R*_(*a*) and *μ*_*L*_(*a*) ≤ *ω*_*L*_(*a*) ≤ *ω*_*R*_(*a*) ≤ *ν*_*R*_(*a*)). In this case we have that

(12) max⁡{|μL(a)−νR(a)|,|μR(a)−νL(a)|} =|μL(a)−νR(a)| ≤|μL(a)−ωR(a)|+|ωL(a)−νR(a)| ≤max⁡{|μL(a)−ωR(a)|,|μR(a)−ωL(a)|}  +max⁡{|ωL(a)−νR(a)|,|ωR(a)−νL(a)|}.

*
Case  3 ( μ*_*L*_(*a*) ≤ *ν*_*L*_(*a*) ≤ *ν*_*R*_(*a*) ≤ *μ*_*R*_(*a*) and *ω*_*L*_(*a*) ≤ *μ*_*L*_(*a*)). In this case we have that

(13) max⁡{|μL(a)−νR(a)|,|μR(a)−νL(a)|} ≤|μL(a)−νR(a)| ≤|ωL(a)−μR(a)| ≤max⁡{|μL(a)−ωR(a)|,|μR(a)−ωL(a)|}  +max⁡{|ωL(a)−νR(a)|,|ωR(a)−νL(a)|}.

*Case  4 ( μ*_*L*_(*a*) ≤ *ω*_*L*_(*a*) ≤ *ω*_*R*_(*a*) ≤ *ν*_*L*_(*a*) ≤ *ν*_*R*_(*a*) ≤ *μ*_*R*_(*a*)). If max⁡{|*μ*_*L*_(*a*) − *ν*_*R*_(*a*)|, |*μ*_*R*_(*a*) − *ν*_*L*_(*a*)|} = |*μ*_*L*_(*a*) − *ν*_*R*_(*a*)|, then we have that

(14) max⁡{|μL(a)−νR(a)|,|μR(a)−νL(a)|} =|μL(a)−νR(a)| =|μL(a)−ωL(a)|+|ωL(a)−νR(a)| ≤|μL(a)−ωR(a)|+|ωL(a)−νR(a)| ≤max⁡{|μL(a)−ωR(a)|,|μR(a)−ωL(a)|}  +max⁡{|ωL(a)−νR(a)|,|ωR(a)−νL(a)|}.

Otherwise, we have that

(15) max⁡{|μL(a)−νR(a)|,|μR(a)−νL(a)|} =|μR(a)−νL(a)| ≤|μR(a)−ωL(a)| ≤max⁡{|μL(a)−ωR(a)|,|μR(a)−ωL(a)|}  +max⁡{|ωL(a)−νR(a)|,|ωR(a)−νL(a)|}.

*Case  5 ( μ*_*L*_(*a*) ≤ *ν*_*L*_(*a*) ≤ *ω*_*L*_(*a*) ≤ *ω*_*R*_(*a*) ≤ *ν*_*R*_(*a*) ≤ *μ*_*R*_(*a*)). In this case we have that

(16) $$\begin{array}{c} \left|\mu_{L}\left(a\right)-\nu_{R}\left(a\right)\right|\leq \left|\mu_{L}\left(a\right)-\omega_{R}\left(a\right)\right|+\left|\omega_{L}\left(a\right)-\nu_{R}\left(a\right)\right|,\\ \left|\mu_{R}\left(a\right)-\nu_{L}\left(a\right)\right|\leq \left|\omega_{L}\left(a\right)-\nu_{R}\left(a\right)\right|+\left|\omega_{R}\left(a\right)-\nu_{L}\left(a\right)\right|. \end{array}$$

Consequently, we have that

(17) max⁡{|μL(a)−νR(a)|,|μR(a)−νL(a)|} ≤max⁡{|μL(a)−ωR(a)|,|μR(a)−ωL(a)|}  +max⁡{|ωL(a)−νR(a)|,|ωR(a)−νL(a)|}.

*Case  6 ( μ*_*L*_(*a*) ≤ *ω*_*L*_(*a*) ≤ *ν*_*L*_(*a*) ≤ *ν*_*R*_(*a*) ≤ *ω*_*R*_(*a*) ≤ *μ*_*R*_(*a*)). In this case we have that

(18) $$\begin{array}{c} \left|\mu_{L}\left(a\right)-\nu_{R}\left(a\right)\right|\leq \left|\mu_{L}\left(a\right)-\omega_{R}\left(a\right)\right|,\\ \left|\mu_{R}\left(a\right)-\nu_{L}\left(a\right)\right|\leq \left|\mu_{R}\left(a\right)-\omega_{L}\left(a\right)\right|. \end{array}$$

Consequently, we have that

(19) max⁡{|μL(a)−νR(a)|,|μR(a)−νL(a)|} ≤max⁡{|μL(a)−ωR(a)|,|μR(a)−ωL(a)|}  +max⁡{|ωL(a)−νR(a)|,|ωR(a)−νL(a)|}.

From what is proved above, we can get that

(20) d(μ,ν)=||μ−ν||=sup⁡0≤a≤1max⁡{|μL(a)−νR(a)|,|μR(a)−νL(a)|}≤sup⁡0≤a≤1max⁡{|μL(a)−ωR(a)|,|μR(a)−ωL(a)|} +sup⁡0≤a≤1max⁡{|ωL(a)−νR(a)|,|ωR(a)−νL(a)|}=||μ−ω||+||ω−ν||=d(μ,ω)+d(ω,ν).

(iv)  Since *μ* ∉ ℝ, there exists *a*_0_ ∈ [0,1] such that *μ*_*R*_(*a*_0_) − *μ*_*L*_(*a*_0_) > 0. Thus we have that

(21) d(μ,μ)=||μ−μ||=sup⁡0≤a≤1|μL(a)−μR(a)|>μR(a0)−μL(a0)>0.

In order to induce a metric which is compatible with the norm, we consider the quotient space of fuzzy numbers. It is very natural to define a function ||·|| : *ℱ*/*𝒮* → ℝ as

(22) $$\begin{array}{c} ||\langle \mu \rangle ||=\underset{\nu \in \langle \mu \rangle }{\inf}||\nu ||, \end{array}$$

for each 〈*μ*〉∈*ℱ*/*𝒮*.

**Theorem 9**

The function ||·|| in (22) is a norm on *ℱ*/*𝒮*.

**Proof**

(i)  It is obvious that ||〈*μ*〉|| ≥ 0 for all *μ* ∈ *ℱ* and ||〈*μ*〉|| = 0 if 〈*μ*〉 = 〈0〉. Conversely, if 〈*μ*〉≠〈0〉, from Lemma 5, it follows that the midpoint function *μ*_*M*_ ≠ 0_*M*_. Thus we have

(23) ||〈μ〉||=inf⁡ν∈〈μ〉||ν||=inf⁡ν∈〈μ〉(sup⁡0≤a≤1max⁡{|νL(a)|,|νR(a)|})≥inf⁡ν∈〈μ〉(sup⁡0≤a≤1|νL(a)+νR(a)|2)=sup⁡0≤a≤1|μM(a)|>0.

(ii)  For all 〈*μ*〉∈*ℱ*/*𝒮* and *b* ∈ ℝ, we have

(24) ||b〈μ〉||=||〈bμ〉||=inf⁡ν∈〈bμ〉||ν||=inf⁡bν′∈〈bμ〉||bν′||=inf⁡ν′∈〈μ〉||bν′||=|b|||〈μ〉||.

(iii)  For all 〈*μ*〉, 〈*ν*〉∈*ℱ*/*𝒮*, we have that

(25) ||〈μ〉+〈ν〉||=||〈μ+ν〉||=inf⁡ω∈〈μ+ν〉||ω||≤inf⁡μ′∈〈μ〉,ν′∈〈ν〉||μ+ν||≤inf⁡μ′∈〈μ〉,ν′∈〈ν〉(||μ||+||ν||)≤inf⁡μ′∈〈μ〉||μ||+inf⁡ν′∈〈ν〉||ν||=||〈μ〉||+||〈ν〉||.

We conclude that ||·|| is a norm on *ℱ*/*𝒮*.

Now we show that the function *ρ* : *ℱ*/*𝒮* × *ℱ*/*𝒮* → ℝ induced by ||·|| as *ρ*(〈*μ*〉, 〈*ν*〉) = ||〈*μ* − *ν*〉|| is exactly a metric on *ℱ*/*𝒮*.

**Theorem 10**

The function *ρ* is a metric on *ℱ*/*𝒮*.

**Proof**

(i)  By the definition of *ρ*, it is obvious that *ρ*(〈*μ*〉, 〈*ν*〉) ≥ 0, for any 〈*μ*〉, 〈*ν*〉∈*ℱ*/*𝒮*. If *ρ*(〈*μ*〉, 〈*ν*〉) = 0, then ||〈*μ* − *ν*〉|| = 0. Thus by Theorem 9, we get 〈*μ*〉 = 〈*ν*〉. In addition, by Lemma 2 we have *ρ*(〈*μ*〉, 〈*μ*〉) = ||〈*μ* − *μ*〉|| = 0.

(ii)  For all 〈*μ*〉, 〈*ν*〉∈*ℱ*/*𝒮*, we have that

(26) ρ(〈μ〉,〈ν〉)=||〈μ−ν〉||=||(−1)〈ν−μ〉||=||ν−μ||=ρ(〈ν〉,〈μ〉).

(iii)  For any 〈*μ*〉, 〈*ν*〉, 〈*ω*〉∈*ℱ*/*𝒮*, we have

(27) ρ(〈μ〉,〈ν〉)=||〈μ−ν〉||=||〈μ−ω+ω−ν〉|| ≤||〈μ−ω〉||+||〈ω−ν〉||=ρ(〈μ〉,〈ω〉)+ρ(〈ω〉,〈ν〉).

We conclude that *ρ* is a metric on *ℱ*/*𝒮*.

## 4. Conclusions

In this present investigation, we studied the norm induced by the supremum metric *d*_*∞*_ on the space of fuzzy numbers. And then we proposed a method for constructing a norm on the quotient space of fuzzy numbers. This norm is very natural and works well with the induced metric on *ℱ*/*𝒮*. The works in this paper enable us to study the fuzzy numbers in the new environment. We hope that our results in this paper may lead to significant, new, and innovative results in other related fields.
